# LSCplus: a fast solution for improving long read accuracy by short read alignment

**DOI:** 10.1186/s12859-016-1316-y

**Published:** 2016-11-09

**Authors:** Ruifeng Hu, Guibo Sun, Xiaobo Sun

**Affiliations:** 1 Beijing Key Laboratory of Innovative Drug Discovery of Traditional Chinese Medicine (Natural Medicine) and Translational Medicine, Beijing, China; 2 Institute of Medicinal Plant Development, Chinese Academy of Medical Sciences & Peking Union Medical College, 151 Malianwa North Road, Haidian District, Beijing, 100193 People’s Republic of China; 3 Key Laboratory of Bioactive Substances and Resource Utilization of Chinese Herbal Medicine, Ministry of Education, Beijing, China; 4 Key Laboratory of the Efficacy Evaluation of Chinese Medicine against Glycolipid Metabolism Disorder Disease, State Administration of Traditional Chinese Medicine, Beijing, China

**Keywords:** SMRT sequencing, RNA-seq, Error correction, Time-consumption

## Abstract

**Background:**

The single molecule, real time (SMRT) sequencing technology of Pacific Biosciences enables the acquisition of transcripts from end to end due to its ability to produce extraordinarily long reads (>10 kb). This new method of transcriptome sequencing has been applied to several projects on humans and model organisms. However, the raw data from SMRT sequencing are of relatively low quality, with a random error rate of approximately 15 %, for which error correction using next-generation sequencing (NGS) short reads is typically necessary. Few tools have been designed that apply a hybrid sequencing approach that combines NGS and SMRT data, and the most popular existing tool for error correction, LSC, has computing resource requirements that are too intensive for most laboratory and research groups. These shortcomings severely limit the application of SMRT long reads for transcriptome analysis.

**Results:**

Here, we report an improved tool (LSCplus) for error correction with the LSC program as a reference. LSCplus overcomes the disadvantage of LSC’s time consumption and improves quality. Only 1/3–1/4 of the time and 1/20–1/25 of the error correction time is required using LSCplus compared with that required for using LSC.

**Conclusions:**

LSCplus is freely available at http://www.herbbol.org:8001/lscplus/. Sample calculations are provided illustrating the precision and efficiency of this method regarding error correction and isoform detection.

**Electronic supplementary material:**

The online version of this article (doi:10.1186/s12859-016-1316-y) contains supplementary material, which is available to authorized users.

## Background

The transcriptomes of organisms are complex, and transcriptome-wide studies on global RNA have been an area of focus for understanding gene expression diversity and functional processes in the post-genomic era [1, 2]. Alternative splicing is essentially universal in organisms with multi-exon genes [3]. A single multi-exon gene may produce several mRNA and protein isoforms that serve various functions [4–6]. Identifying and quantifying these transcript isoforms is important [7–10]. Due to their relatively short lengths, the reads are unable to cover an entire RNA molecule, which leads to difficulties in identifying transcript isoforms [11, 12].

The single molecule, real time (SMRT) sequencing technology of Pacific Biosciences (PacBio) has advantages for transcriptome sequencing [13–15]. SMRT sequencing is appreciated for its ability to produce continuous long reads with an average length of more than 10,000 bp, which enables the acquisition of transcripts from end to end. Furthermore, SMRT sequencing has no sequence preference and can provide more information than other technologies on transcript isoforms. Although these continuous long reads can be used to capture large isoform fragments or even full-length isoform transcripts, there are high error rates in these long reads (nearly 15 %) [16]. Most errors are base deletions or insertions, particularly in the polymer segments. These errors occur randomly. PacBio has used an approach called “circular consensus sequencing” (CCS) to improve the sequencing accuracy; however, CCS increases the sequencing depth, which adds huge costs and limits the sizes of reads (< 1.5 kb). Hybrid sequencing has been used for genome sequencing [17–19], but in the field of transcriptome sequencing, there are few tools that have been developed for this task [20].

Au et al. wrote an error correction tool named LSC [20] in the Python language that combines the strengths of next-generation sequencing (NGS) accurate short reads (SRs) and PacBio long reads (LRs) for the task of isoform assembly from RNA-seq data. LSC can perform correction well; however, it has some shortcomings. In LSC, a homopolymer compression (HC) transformation strategy is applied to enable accurate SR-LR alignment, but in the error correction step, it does not account for the points at which the HC count is 1, and mismatched bases are not corrected. Furthermore, LSC is too time-consuming to run in a typical laboratory. Many users have reported that LSC has computing resource requirements that are too intensive for most laboratory and research groups, which severely limits the application of SMRT long reads for transcriptome analysis. This program must be run in a specific biological service center or data analysis center equipped with high-performance computing equipment [17]. In this project, we referenced the LSC program and developed a faster tool, named LSCplus (LSC^+^), written in the C++ and Python languages, for better performance in sequence correction. LSCplus lacks the shortcomings of LSC and is 3-4-fold faster than LSC. Remarkably, it requires only 1/20–1/25 of the time required by LSC for the main step of error correction. A sample calculation provided in this paper demonstrates the precision and efficiency of this method. LSCplus is freely available at http://www.herbbol.org:8001/LSCplus.

## Implementation

The workflow of LSCplus includes SR & LR HC transformation, SR quality control, SR-LR alignment, and error correction and is based on LSC. LSCplus uses different SR&LR homopolymer compression (HC) transformation and error correction from those of LSC.

### SR & LR HC transformation

Due to the sequencing theory limitations of the PacBio platform, there is a high error rate in the results. Most of these errors are base deletions or insertions, particularly in polymer segments, and they occur randomly. To eliminate mistakes consisting of an uncertain number of a particular base in polymer segments, an HC transformation strategy was applied to increase the sensitivity of SR-LR alignment.

**Fig. 1:**
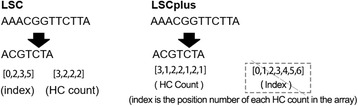
HC results of LSC and LSCplus. LSC records the compressed positions and corresponding HC counts. LSCplus records all of the ‘HC’ counts corresponding to the compressed sequence. In LSCplus, “AAACGGTTCTTA” will be compressed to “ACGTCTA”, and the HC Counts Array for the compressed read is HC = [3,1,2,2,1,2,1]. The index array will be [0,1,2,3,4,5,6], which represents the position numbers of HC count in the array as well as the position numbers of bases in the compressed read; once the HC array is constructed, the indexes are set, such that the index array is not needed

Compression has been shown to lead to little sacrifice in sensitivity in mapping, and the compressed sequences retain the ability to allow identification of a genomic location. Due to speed considerations, we used multi-thread programming. First, we divided the SR file into several small files according to the thread number set in the configuration file. Each thread handles a small separate file. In the process of homopolymer compression (HC), the homopolymer sequences in LRs and SRs are replaced by a single base of the same type. The compressed sequences are stored in *.cps files (compression), and the HC count is recorded in a corresponding *.hcc file (HC Count). The record in our *.hcc file is different from the record in LSC, as we record not only the compressed positions but also the uncompressed positions, which means that the HC count is 1 (Fig. 1).

### SR quality control

As in LSC, the poor-quality sequences are excluded from the cps and idx files. Examples include extremely short reads with lengths less than 40 bp and reads with too many ‘N’s, which may cause alignment errors. Users can change these values in the configuration file.

### SR-LR alignment

In LSCplus, Bowtie2 is embedded as the default alignment program; therefore, users do not need to install Bowtie2 or be concerned about installation errors. Bowtie2 [21] is an ultrafast alignment program that outputs sam files, from which we can obtain detailed alignment information. Bowtie2 is also now the default alignment program in LSC. The mapping quality can be controlled by Bowtie2 via arguments set by the user. In addition, in LSCplus, there is an argument (max_error_rate) to limit the LR-SR alignment error rate. max_error_rate is the maximum error rate percentage allowed for accepting a compressed LR-SR alignment. If the error rate is greater than the max_error_rate value, the SR will be dropped.

### Error correction

**Fig. 2:**
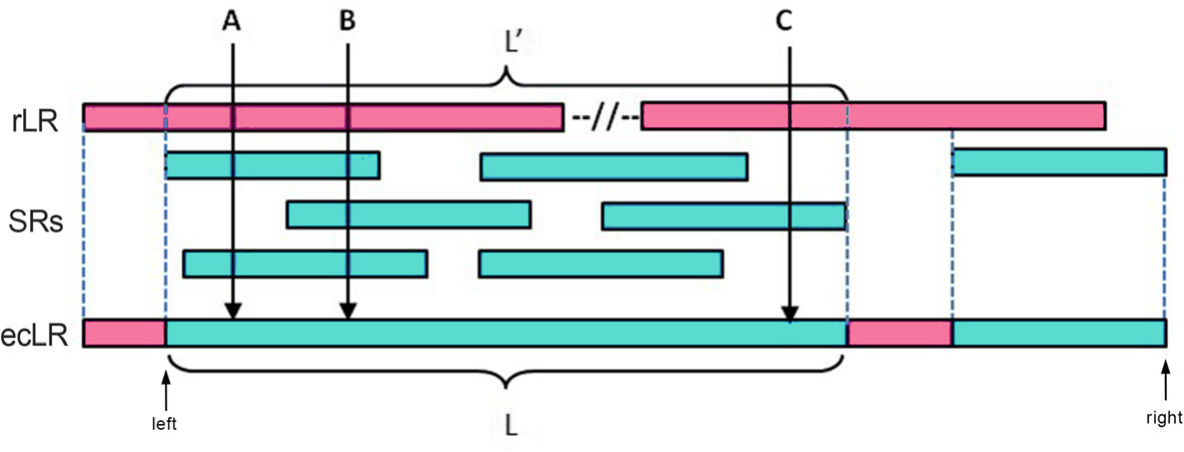
Process of error correction. Here, rLR (raw Long Read) is the raw long read for correction, and ecLR (error-corrected Long Read) corresponds to rLR. There may be many short reads mapped to rLR. L’ is the length of a region covered by the SRs, and L is the length of a corrected region of an ecLR corresponding to SRs mapped to the rLR. Due to the indels in rLRs, L may not be equal to L’. Positions A, B, and C are three example positions covered by 2 SRs, 3 SRs, and 1 SR, respectively. The subsequence between the left-most SR-covered point (*left*) and the right-most SR-covered point (*right*) of the ecLR is defined as lrLR (left point-right point Long Read), which is stored in the file corrected_LR.fa, and the full length ecLR is written in the corrected file_LR_full.fa. The uncovered regions are retained. (Pink indicates that the bases came from rLR; light green indicates that the bases came from mapped short reads)

We parsed the sam files, retrieved the useful information and generated the LR-SR mapping file. From the LR-SR mapping file, we can obtain a layout of each compressed LR with the aligned compressed SRs (Fig. 2). For uncovered regions of a raw long read (rLR), the rLR sequence is retained in an error-corrected long read (ecLR). For rLR regions covered by SRs, we assume that the SRs are correct. In a specific position, one of the covered bases with the corresponding idx information is selected, and we calculate the frequency of this base in the entire candidate base list. The frequency of the selected base can be set in the configuration file as the Short-Reads Coverage Frequency (SCF) value (the default is 60, which means that the frequency of the selected base should be more than 60 %). If no base is selected, the base with the highest frequency will be selected, and then the selected base will be used as the correct base and appended to the end of the ecLR (Additional file 1, Pseudo code). For example, in Fig. 2, position A is covered by two compressed SR sequences, and one of the two bases is selected as the corrected base with the corresponding HC count. Position B is covered by three compressed SR sequences; therefore, the correct base with its HC count is the base in one of these sequences (if they are different from each other) or the most frequent base. Position C is covered by only one compressed SR sequence; therefore, it is the correct sequence. Thus, if a position is only covered by two or three SRs that differ from each other, it is impossible to tell which one is real. If such a case occurs, LSCplus selects the first base in the list. By increasing the coverage depth, more true positives are obtained, and the true positive rate is increased. In most cases, the base callings in a specific position are the same. Position B of the SRs may in all cases be base T. Thus, we do not use the parameter Short-Reads Coverage depth (SCD) introduced in LSC or set SCD to 0, which means that all mapped SRs are used to correct the rLR. If the mapped SRs exceed the end of the rLR, we use the SRs to extend the LR.

In this step, the decompression is performed while selecting the base. Error correction is the last step and the core step. We have adopted a different strategy from that in LSC (Pseudo code, Additional file 1: Figures S1 and S2). In LSC, deletion and insertion errors are separated in the error correction step, and for a given error correction position, LSC loops through all of the candidates. LSCplus uses the SCF value to determine the suitable bases; this loop will occur only once or twice in the vast majority of cases. For example, suppose that the candidates list is *N* = [‘A’,‘A’,‘A’,‘C’,‘A’,‘G’] for an error correction position (SCF = 60). First, we obtain the list length (candidate number, which is 6 in this example). Then, the first ‘A’ is selected and the number of times ‘A’ appears in the list is calculated (4). 4/6 ≈ 0.667 > 0.6 = SCF/100; therefore, ‘A’ is selected. This only loops once. If the candidates list is [‘G’,‘A’,‘A’,‘A’,‘C’,‘A’], it loops twice; if SCF = 10, it also loops once. SCF is a parameter that balances speed and accuracy. Loops are the most time-consuming operation in a program, particularly nested loop structures; we use this method to reduce loop time to obtain greater speed. We do not use sort in advance because the first item after sorting is always ‘A’ followed by ‘C’ > ‘G’ > ‘T’ in the candidates list and the bases needed in error positions are random. Random selection is better than sorted selection. We also consider both deletion and insertion together to reduce the loop times and loop nesting levels.

## Results

LSCplus is designed for RNA-seq analysis. We addressed some of the shortcomings of LSC to improve this method. We tested LSCplus using the example data sets provided by the official LSC website (http://www.healthcare.uiowa.edu/labs/au/LSC/files/example.rar), which are RNA-seq data on the human brain. There are 57,244 long reads in the LR file, with an average length of ~798 bp, and there are 1,000,000 single-end short reads in the SR file. We applied LSCplus to these data sets and obtained 31,133 ecLRs.

### Quality of ecLRs

LSCplus outputs two files. The full-length ecLRs are stored in the corrected_LR_full.fa, and the subsequence between the left-most SR-covered point and the right-most SR-covered point of the ecLR (lrLR) (Fig. 2, left point to right point) is written into corrected_LR.fa. The lrLRs from LSCplus and LSC were mapped to the human genome (hg38 and GRCh38, respectively) using BLAT [22] (http://hgdownload.soe.ucsc.edu/admin/exe/linux.x86_64/blat/blat). We conducted a sequence identity measurement, which is defined as

$$ SI=\frac{the\  number\  of\  matches}{length\  of\  read} $$

LSCplus outputs the same number of lrLRs as does LSC. Here, 30,910 lrLRs were mapped to the reference genome, whereas the corresponding number for LSC was 30,850. In total, 71.40 % of the mapped lrLRs had a sequence identity greater than or equal to 0.9, which was nearly equal to the percentage observed for LSC (71.38 %); the percentages of SI values greater than or equal to 0.8 and 0.7 (81.56 and 84.39 %, respectively) are slightly higher than those of LSC (81.43 and 84.28 %, respectively). In addition, the average length of LSCplus-lrLRs was 663 bp, which was longer than for that for LSC (Table 1).

**Table 1:** Quality of lrLRs and comparison between LSCplus (SCF = 60) and LSC (SCD = 20)

		LSCplus	LSC_1_beta	LSC_2	Raw_Data (rLR)
Output lrLR number		44,497	44,497	44,491	\
Mapped lrLR number		44,216	44,112	44,110	16,947 (rLR)
Average length		601.46bp	600.36bp	600.61bp	\
Time consumed	Total	185s	832s	1h 33m 21s	\
	EC^a^ step	23s	546s	\	\
Sequence identity	SI >0.9	71.40 %	71.38 %	71.34 %	0.84 %
	SI >0.8	81.56 %	81.43 %	81.44 %	19.02 %
	SI >0.7	84.39 %	84.28 %	84.37 %	37.66 %

^a^ *EC* Error correction

**Fig. 3:**
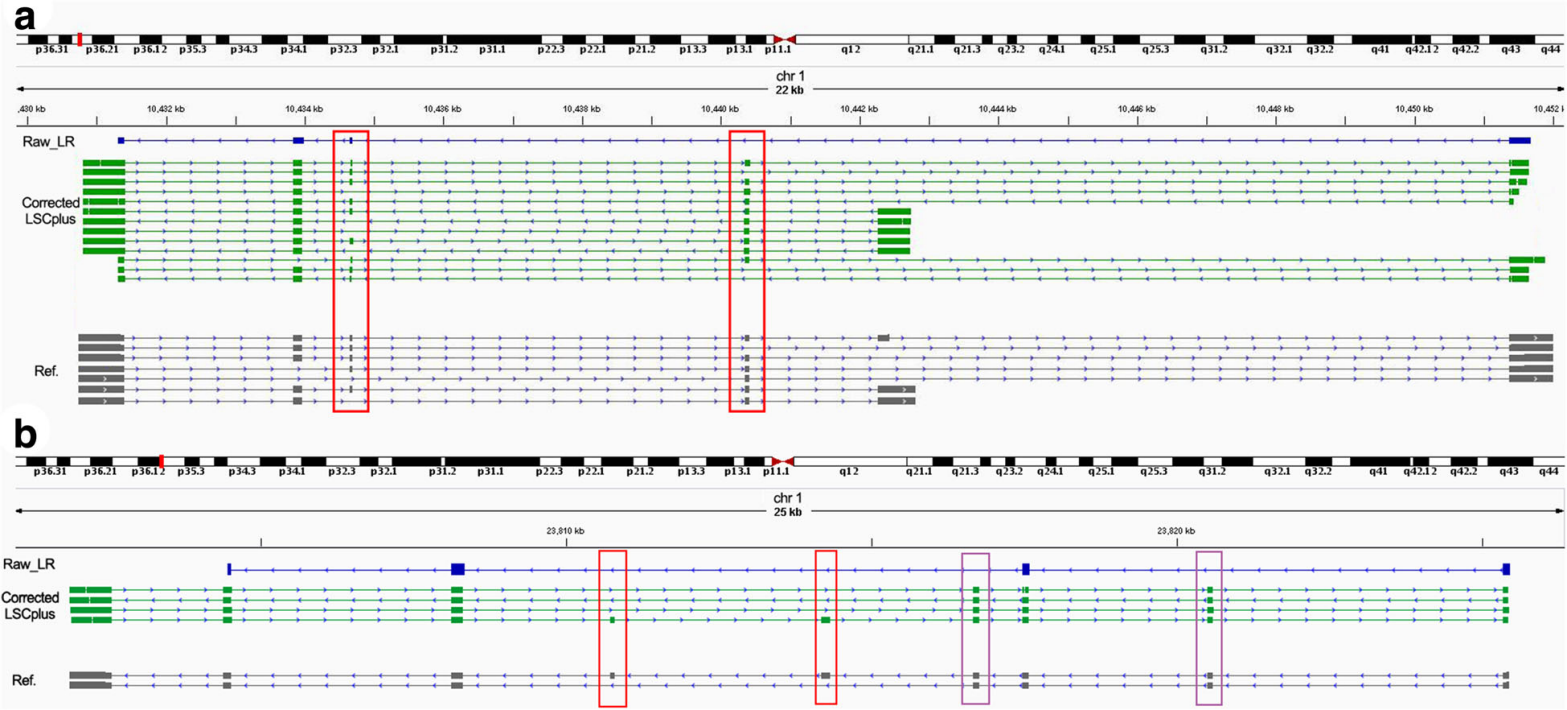
Two local details of the alignments of raw LRs and LSCplus-corrected LRs. (**a**) Two positions of isoform identification. (**b**) Two positions of isoform identification and Two positions of exons recovery. Error correction of RNA-seq data provides more accurate mapping of transcripts. A genome browser view of transcriptome alignments using uncorrected (*blue*) and corrected (*green*) PacBio reads. Color blocks represent the exons. Before correction, only one potential transcript isoform was detected with any exons missing (*indicated with purple rectangles*), and after correction, the corrected sequences matched the reference annotations end to end with no exons missing. As a result, the isoforms (*indicated with red rectangles*) at the displayed reference locus in the reference annotation were confirmed by corrected PacBio RNA-seq reads

Many of the PacBio reads represented close to full-length transcripts. However, the exon structure was not evident before error correction. As shown in Fig. 3, there are two local details of the alignments of raw LRs and LSCplus-corrected LRs. Figure 3a shows the APITD1-CORT gene, and Fig. 3b shows the HMGCL gene on chromosome 1. Error correction of RNA-seq data provides more accurate mapping of transcripts. The genome browser view of transcriptome alignments shows uncorrected (blue) and corrected (green) PacBio reads of human brain cerebellum polyA RNA corrected by Illumina’s Human Body Map 2.0 project SR data (GSE30611). The splice-aware aligner BLAT [22] was used to align PacBio reads to the genome. Long gaps in the alignment correspond to introns in the PacBio reads but not the reference genome. Color blocks represent the exons. In Fig. 3, “exons recovery” is indicated by purple rectangles and “isoform identification” is indicated by red rectangles. Figure 3a shows the isoform identification, indicated with red rectangles, and the isoforms at the displayed reference locus in the reference annotation were confirmed by corrected PacBio RNA-seq reads. As shown in Fig. 3b, the two isoforms were identified (red rectangles) after correction, whereas one isoform was missing before correction. Figure 3b shows that before correction, only one potential transcript isoform was detected with any exons missing (indicated with purple rectangles); after correction, the corrected sequences matched the reference annotations end to end with no exons missing. Exons missed from raw long reads were recovered from error-corrected reads (Fig. 3b, purple rectangles).

In LSCplus, the bases with HC = 1 are considered for correction, which is not done in LSC. The base numbers of the different error types were counted: (1) example dataset: Base number of long reads: 45,717,936; Insert Error: 925,887; Delete Error: 339,718; Mismatch Error: 1,087,287; HC Error: 240,828 (HC = 1: 75,161); (2) human brain dataset: Base number of long reads: 138,156,931; Insert Error: 3,708,177; Delete Error: 1,251,726; Mismatch Error: 5,084,895; HC Error: 1,772,516 (HC = 1: 352,271). The results show that error bases with HC = 1 should be considered. The ability to correct bases with HC = 1 is one of the main features of LSCplus.

### Time consumption

The running time of LSC is substantially reduced compared with that of PacBioToCA; however, LSC remains too time-consuming to run in a typical laboratory or research setting. For a whole-transcriptome dataset, an LSC run typically lasts several weeks to a few months; thus outsourcing the job to a specific analysis center or high-performance calculation department is necessary. We tested LSCplus and LSC (v1_beta and v2) using example data sets on a server with four cores (Intel(R) Xeon(R) CPU E5-2620, 2.00 GHz, 32 GB RAM) equipped with CentOS 6.5 using 20 threads. All programs were repeated 10 times, and we obtained the average time span. LSCplus consumed an average time of 185 s for a complete run and 23 s for the last core step. LSC consumed an average time of 764 s for a complete run and 496 s for the last core step. LSCplus sharply reduced the running time to 1/3–1/4 that of LSC. The error correction step is the most time-consuming step in LSC and accounts for nearly half of the total running time. Notably, LSCplus only uses 1/20–1/25 the time of LSC for the error correction step. LSC2 performs worst among LSCplus, LSC1_beta and LSC2. LSC2 required more than 90 min (Table 1).

## Comparison against other error correction tools for PacBio long reads

We compared LSCplus against other error correction tools for PacBio long reads using human brain transcriptomic data.

### Installation and parameter settings

PubMed was searched to identify software published before 3rd September 2015 using the following term: ‘error correction pacbio’, resulting in the retrieval of 8 papers addressing 4 software platforms: LSC [20], PacBioToCA [18], proovread [17] and LoRDEC [19]. We downloaded the latest versions of the software and attempted to install LSC (v1_beta; Bowtie2 is the default aligner; the latest version of LSC is LSC2, but it requires more running time than v1_beta for the same dataset), PacBioToCA (v8.1), proovread (v2.12) and LoRDEC (v0.5) on our system (CentOS 6.5) to evaluate their performance. All of these platforms depend on third-party programs (publicly used or self-developed programs or libraries), and proovread failed to be installed successfully. Furthermore, proovread and LoRDEC provide many parameter settings that have great impacts on the output. Many of these parameters are difficult to understand for users who are unskilled in mathematics or computer science (Table 2).

**Table 2:** Installation information and parameter settings of the different programs

Tools	Dependencies	Parameters	Complexity
LSCplus	Bowtie2 (integrated)	Few and easy to understand	A compressed file. Download, uncompress and run.
LSC	Aligner (not integrated) (bowtie2/bwa/novoalign/razers3)	Few and easy to understand	Install the Aligner and set the PATH. A compressed file. Download, uncompress and run.
PacBioToCA	AMOS package (AMOS depends on many other programs)	Few and easy to understand	Source code or binaries. Strict requirements regarding the format of input content.
proovread	Perl 5.10 or later, Log::Log4perl, Blast-2.2.24+ or later, samtools-1.1 or later, BLASR (integrated)	Many and specialized	Source code needs to be compiled and depends on many other libraries.
LoRDEC	gcc version 4.5 or newer, Boost C++ libraries, GATB Core library.	Many and specialized	Source code needs to be compiled and depends on many other libraries. The steps are cumbersome and error-prone.

### Quality and time consumption

We evaluated the correction efficiency of LSCplus compared with that of the existing pipelines LSC (v1_beta and v2), PacBioToCA (v8.1, hybrid-correction) and LoRDEC (v0.5) using two real biological datasets, one library of long PacBio reads and one library of RNA-seq short reads: (1) human brain cerebellum polyA RNA processed to enrich for full-length cDNA for the PacBio RS platform under C2 chemistry conditions as LR data [20] (174,246 PacBio long reads, http://www.healthcare.uiowa.edu/labs/au/LSC/files/human_cerebellum_PacBioLR.zip) and (2) human brain data from Illumina’s Human Body Map 2.0 project (GSE30611, 64,313,204 single-end reads, 75 bp) as SR data. All of the programs were tested on a server with eight cores (Intel(R) Xeon(R) CPU E7- 8837 @ 2.67 GHz, 64 GB RAM) equipped with CentOS 6.5 using 20 threads. After applying the pipelines LSCplus (v2.25), LSC (v1_beta and v2), PacBioToCA (v8.1, hybrid-correction) and LoRDEC (v0.5), we obtained the output and summarized the results. Each program was tested at least three times on these data. For LoRDEC, as several parameters impact the correction process, we evaluated this program by setting different parameter values (Tables 3 and 4).

**Table 3:** Results from running LSCplus, LSC and PBcR

		LSCplus	LSC (v1_beta)	LSC (v2)	PacBioToCA
Output lrLR number		155,399	155,398	159,168	109,078
Mapped lrLR number^a^		155,305	155,286	159,000	108,170
Average length of lrLRs		832.21 bp	831.30 bp	731.09 bp	101.28 bp
Time consumed		1 h 52 min	6 h 52 min	42 h 35 min	58 h 57 min
Sequence identity	SI ≥ 0.9	73.63 % (114,351)	71.78 % (111,464)	74.62 % (118,654)	86.94 % (94,043)
	SI ≥ 0.8	78.93 % (122,582)	78.59 % (122,039)	80.13 % (127,410)	92.49 % (100,047)
	SI ≥ 0.7	81.22 % (126,138)	81.17 % (126,045)	82.37 % (130,973)	95.68 % (103,497)

^a^Number of lrLRs that mapped to hg38

**Table 4:** Results of running LoRDEC under different parameter values

Parameters					Output lrLRs number	Mapped lrLRs number	Average length of lrLRs	Time consumed	Sequence identity		
K	Solid	Target	Branch	Error rate					SI ≥ 0.9	SI ≥ 0.8	SI ≥ 0.7
19	3	5	200	0.4	164,893	152,778	736.91 bp	47 min	52.06 % (79,533)	71.20 % (108,778)	77.20 % (117,949)
19	3	10	2000	0.4	164,893	153,341	737.85 bp	4 h 46 min	50.97 % (78,152)	68.91 % (105,671)	74.74 % (114,613)
19	3	15	200	0.4	164,893	153,222	737.25 bp	2 h 30 min	50.52 % (77,417)	68.76 % (105,363)	74.66 % (114,390)
19	3	10	200	0.4	164,893	153,061	737.06 bp	1 h 42 min	51.36 % (78,619)	69.95 % (107,080)	75.85 % (118,118)
19	3	5	1000	0.4	164,893	152,936	737.36 bp	1 h 20 min	52.24 % (79,895)	70.95 % (108,519)	76.88 % (117,583)
19	3	10	5000	0.4	164,893	153,436	738.11 bp	10 h 2 min	50.67 % (77,760)	68.43 % (104,998)	74.33 % (113,896)
21	3	5	200	0.4	160,363	147,928	722.04 bp	1 h 24 min	61.44 % (90,932)	75.62 % (111,904)	79.75 % (118,021)
21	3	10	2000	0.4	160,363	148,100	723.19 bp	4 h 07 min	61.73 % (91,426)	75.23 % (111,422)	79.39 % (117,580)
21	3	15	5000	0.4	160,363	148,114	723.78 bp	16 h 46 min	61.43 % (91,009)	74.64 % (110,589)	78.88 % (116,865)
21	3	15	1000	0.45	160,363	148,080	722.10 bp	5 h 26 min	60.46 % (89,537)	74.23 % (109,933)	78.68 % (116,511)
21	5	15	1000	0.45	159,529	146,754	722.13 bp	4 h 39 min	64.81 % (95,117)	76.73 % (112,617)	80.41 % (118,004)
21	2	15	1000	0.45	161,025	149,055	720.70 bp	6 h 55 min	54.62 % (87,416)	71.25 % (106,202)	76.73 % (114,365)

PacBioToCA presented the worst performance regarding average lrLR length, which negated the advantages of SMRT sequencing and was in conflict with our primary goals. LSC1_beta exhibited similar performance to LSCplus regarding the quality of corrected lrLRs; however, LSCplus required 1/3–1/4 the running time of LSC (v1_beta). Notably, LSC2 obtained higher SI values and higher output and mapped lrLR numbers but required approximately six-fold more time than LSC1_beta and over twenty-fold more time than LSCplus. The average length of the lrLRs was approximately 100 bp shorter than that for LSC1_beta or LSCplus. We ran LoRDEC under different conditions. The average length of the LoRDEC output was approximately 100 bp shorter than that for LSCplus. For a longer average length, LoRDEC must be set with a higher branch value, which results in a longer running time. The output lrLR number and mapped lrLR number were lower than those of LoRDEC; however, the obtained sequence identity was better than all of the outputs of LoRDEC. The outputs of LoRDEC were impacted by the parameter values. These results demonstrate the precision and efficiency of LSCplus.

## Discussion

In eukaryotic organisms, the majority of genes are alternatively spliced to produce multiple transcript isoforms, which dramatically increases the protein-coding potential of a genome. Alternatively, spliced isoforms produced from the same gene can have significantly different and even antagonistic effects. To study gene expression, researchers have examined gene fragments of organisms utilizing next-generation sequencing methods, commonly referred to as RNA-seq. However, short-read RNA-seq cannot span full-length transcripts, making it difficult to accurately characterize the diverse landscape of isoforms.

NGS technology has been a powerful tool in modern biology; however, the relatively short sequence length has limited its application in transcriptome analysis. It is essential to understand the transcriptome to determine the functional elements of the genome and to reveal the molecular constituents of cells and tissues [23]. Great gains have been made using NGS methods; however, these methods also have several drawbacks. First, they require the amplification of source DNA before sequencing, leading to amplification artifacts and biased coverage of the genome related to the chemical-physical properties of the DNA. Second, current technologies produce relatively short reads with median lengths of 100 bp obtained through Illumina sequencing (max. 150 bp) and ~700 bp for 454 sequencing (max. 1,000 bp). Short reads make assembly and related analyses difficult, with theoretical modeling suggesting that decreasing the read length from 1,000 bp to 100 bp can lead to a six-fold or more decrease in continuity. Pacific Biosciences SMRT aims to address the problems outlined above by requiring no amplification and reducing compositional bias, thereby producing long sequences. Single-molecule sequencing instruments can generate multikilobase sequences with the potential to greatly improve genome and transcriptome assembly. Such long read lengths will be beneficial for de novo genome and transcriptome assembly as they have the potential to resolve complex repeats and span entire gene transcripts. However, the instrument generates reads with an average nucleotide accuracy of only 82.1–84.6 %, showing uniformly distributed errors dominated by point insertions and deletions, which obscures alignments between reads and complicates the analysis. Furthermore, increasing the alignment sensitivity of traditional assemblers is computationally unfeasible.

Because the length of single-molecule PacBio reads (ranging from a few hundred bases to several kilobases) obtained from RNA-seq experiments is within the size distribution of most transcripts, PacBio reads will represent full-length or near full-length transcripts. These long reads can therefore greatly reduce the need for transcript assembly (which requires complex algorithms for short reads) and allow confident detection of alternatively spliced isoforms. However, the predominance of indel errors makes the analysis of raw reads problematic [18]. As described in this report, only 0.84 % of the example mRNA reads were aligned to the reference genome by BLAT [22] at >90 % sequence identity. In contrast, for the corrected sequences, the percentage of sequences that aligned with a >90 % identity increased dramatically to more than 70 %.

During the error correction process, if a specific position only covers a few different bases, the program cannot decide which one is real. By increasing the coverage depth, the number of true positives is increased, increasing the true positive rate. In most cases, the base callings in a specific position are the same.

Due to the high error rate in PacBio long reads, hybrid sequencing is needed. However, there are few tools in the field of hybrid error correction for transcriptomes. LSC is used widely and is officially recommended by Pacific Biosciences. Many users report that LSC has computing resource requirements that limit the application of SMRT long reads for transcriptome analysis. To reduce the running time, the original algorithm in the error correction step of LSC was optimized in LSCplus. Algorithm optimization is currently a popular topic in the field of computational science. We used the SCF value to balance the accuracy and speed of LSCplus. For higher accuracy, the SCF can be set to a higher value. We also tested LSCplus under various SCF values. At an SCF value of 100, 71.43 % of all ecLRs had an SI value greater than or equal to 0.9, which is a slightly higher percentage than that obtained with LSC; and the run time of the error correction step was approximately 32 s, which is less than that obtained with LSC (SCD = 20; Table 5). We found that 60 is an appreciated value for SCF. SCD (Short-reads coverage depth) is an argument defined by LSC. It was used to generate the LR-SR alignment file with the expected SR coverage depth of SCD value. The SCD filter is applied to LR segments with an SR coverage greater than the SCD value. At smaller SCD values, more LR segments with lower coverage depth are retained, which increases the running time, with more error positions fixed. At larger SCD values, more LR segments with lower coverage depth are omitted; this increases the reliability of the correction, but more error positions escape the correction. The default value of SCD is 20, which means that LR-SR alignments with a coverage depth less than 20 are neglected for correction. LSCplus uses all of the mapped SRs to perform the correction. Because of the high accuracy of the SRs (>98 %), the SRs are more reliable, and thus, the SCD Value is not needed. The test results also revealed that even when all mapped SRs were used, LSCplus ran faster than LSC with filtered SRs. LSCplus is a fast solution for improving long read accuracy using short read alignments.

**Table 5:** lrLR quality and time consumption under different SCF values

LSCplus			LSC (SCD = 20)	
SCF value	Percentage (SI ≥ 0.9)	Time consumption (Error correction)	Percentage (SI ≥ 0.9)	Time consumption (Error correction)
20	70.57 %	19 s	71.34 %	496 s
40	70.77 %	21 s		
60	71.40 %	23 s		
80	71.43 %	27 s		
100	71.43 %	32 s		

## Conclusion

Isoform identification is one of the most important aspects of transcriptome analysis. Most genes in an organism can express multiple mRNA and protein isoforms that perform specific functions [24, 25]. SMRT sequencing opens the door to many types of downstream analysis with its long-read sequencing, which is useful for de novo assembly. Using long reads, the success rate in obtaining long contigs in sequence assembly is very high. It is easier to obtain long contigs with long reads than with short reads (Fig. 3). The formats of the output files from LSCplus are the same as those from LSC; thus, the results can be used directly in IDP [11], an isoform detection and prediction tool.

However, because the long reads cannot be fully covered by short reads and because there is a high random error rate in long reads, uncovered regions represent the main areas of mismatches. Error correction may produce artifacts due to alignment errors, and the hybrid reads are not truly single-molecule reads [1]. LSCplus is a rapid solution for improving long read accuracy using short read alignments. LSCplus greatly overcomes the disadvantage of LSC’s time consumption and improves quality; therefore, it can be used by most laboratory and research groups. These advantages may facilitate many important discoveries in life science. LSCplus allows users to make full use of the advantages of PacBio long reads. LSCplus is freely available at http://www.herbbol.org:8001/lscplus/. The sample calculations presented in this paper demonstrate the precision and efficiency of this method regarding error correction and isoform detection.

## Additional files

Additional file 1: Pseudo code for LSCplus Error Correction and Flowcharts of the Error Correction Steps in LSCplus and LSC. (PDF 541 kb)
Additional file 2: Detailed description of the operational steps of LSCplus using the PacBio e-coli dataset ERR1274825 and the Illumina e-coli dataset SRR3372215. (PDF 59 kb)

## Abbreviations

CCS: Circular consensus sequencing
EC: Error correction
HC: Homopolymer compression
LR: Long read
NGS: Next-generation sequencing
PacBio: Pacific Biosciences
SCD: Short-reads coverage depth
SCF: Short-Reads Coverage Frequency
SI: Sequence identity
SMRT: Single molecule, real time
SR: Short read
