# Supplementary material for: LSCplus: a fast solution for improving long read accuracy by short read alignment
Source: BMC Bioinformatics. 2016 Nov 9;17:451. doi: 10.1186/s12859-016-1316-y (PMC5103424; doi:10.1186/s12859-016-1316-y)
Supplement: Additional file 1: — Pseudo code for LSCplus Error Correction and Flowcharts of the Error Correction Steps in LSCplus and LSC. (PDF 541 kb) [file 12859_2016_1316_MOESM1_ESM.pdf]

Pseudo code for Error Correction

```
N = ['A', 'A', 'A', 'C', 'A', 'G'];
// N is the candidate list of bases for a position
  i = 0;
// selected from the first one
while (i < Length_of_N) {
            // choose the first candidate
            N_base = N[i];
            //Get the number of the N_base
            N_count = count (N, N_base);
            //Calculate the Frequency of the N_base in N
            Frequency = N_count / total number of all candidates contained in N list;
            //if the Frequency is greater than SCF, then the N_base will be selected and break the loop
            If (Frequency*100 >= SCF) {
                  selected_base _i= i;
                  break;
                  }
            //We note the most frequently appearing base in N; in the case in which no base reaches the SCF value, the most frequent base will be used.
            If (N_count > max_count ){
                  max_count = N_count;
                  selected_base_i = I;
                  }
            i = i+1; //next candidate
      }
      selected_base = N[selected_base_i];
      ecLR.append(selected_base*HC_count)
```

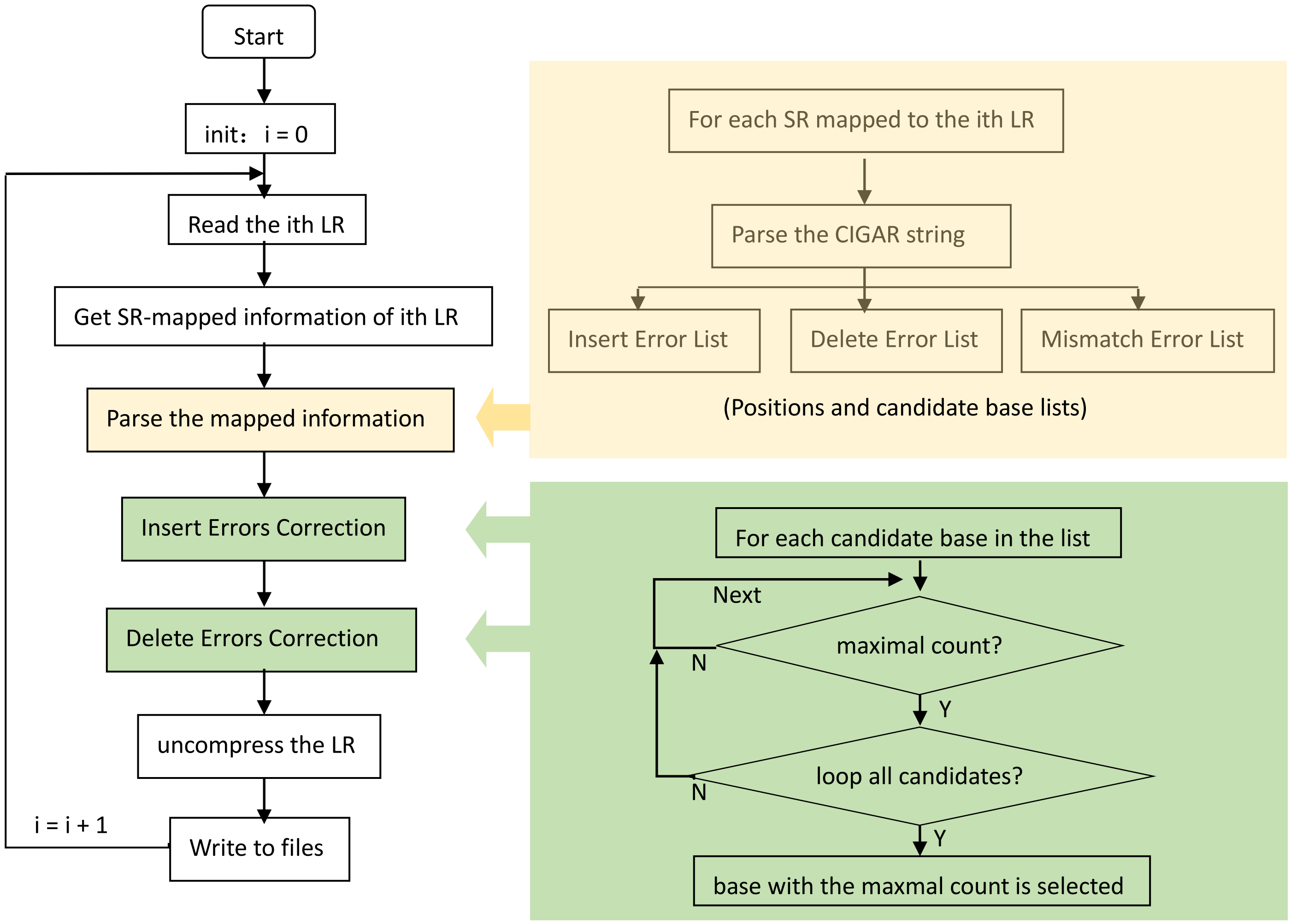

Supplementary Figure 1. Flowchart of Error Correction Step in LSC

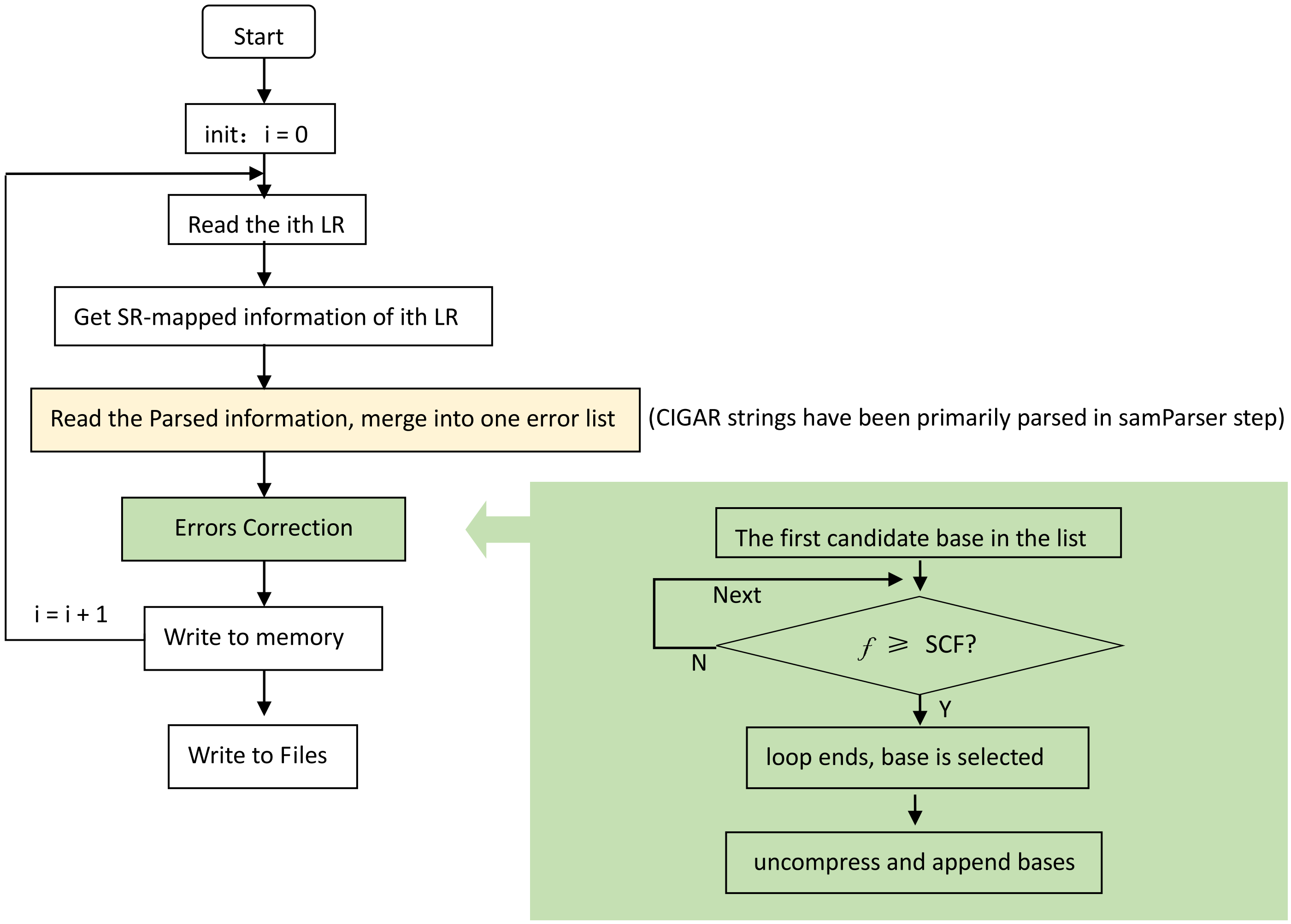

Supplementary Figure 2. Flowchart of Error Correction Step in LSCplus
