# Supplementary material for: LSCplus: a fast solution for improving long read accuracy by short read alignment
Source: BMC Bioinformatics. 2016 Nov 9;17:451. doi: 10.1186/s12859-016-1316-y (PMC5103424; doi:10.1186/s12859-016-1316-y)
Supplement: Additional file 2: — Detailed description of the operational steps of LSCplus using the PacBio e-coli dataset ERR1274825 and the Illumina e-coli dataset SRR3372215. (PDF 59 kb) [file 12859_2016_1316_MOESM2_ESM.pdf]

From the SRA, we downloaded the pacbio e-coli dataset(ERR1274825) and the Illumina e-coli dataset(SRR3372215)

LSCplus is designed for RNA-seq analysis and only FASTA files are supported by the latest LSCplus.
We have developed a toolkit (LSCplus_toolkit) for preparing SR.fa and LR.fa file, and can be downloaded from LSCplus website.

A detailed description of the steps is listed below (***Step 4 and Step 5 are optional, not necessary***):

Steps:
1. Fetch ERR1274825.sra;
    command: fastq-dump ERR1274825
    result: ERR1274825.sra was downloaded;
    (fastq-dump is part of The NCBI SRA Toolkit. http://www.ncbi.nlm.nih.gov/Traces/sra/)
2. Convert ERR1274825.sra to ERR1274825.fastq;
    command: fastq-dump ERR1274825
    result: ERR1274825.fastq is got.
3. Convert ERR1274825.fastq to FASTA format with modified names (the pacbio reads names should be in the format "name/index/1_(length)", where (length) is the length of the read.
    command: python ConverToPacBio_q2a.py ERR1274825.fastq
    result: LR.fa is got.
    (ConverToPacBio_q2a.py is part of LSCplus_toolkit)
4. Align the pacbio reads to the e.coli reference(ecoli_K12) using blasr with the parameters "-sam -bestn 1";*
    command: ./blasr LR.fa ecoli_K12.fasta --sam --bestn 1 --out out.sam --header
    result: out.sam is got
5. Selecte the aligned subsequences for the first 4000 reads;*
    command: python selectTopN.py out.sam 4000 LR.fa
    result: The new LR.fa file is got.
6. Fetch SRR3372215.sra;
    command: fastq-dump SRR3372215
    result: SRR3372215.sra was downloaded.
7. Convert SRR3372215.sra to SRR3372215_1.fastq and SRR3372215_2.fastq;
    command: fastq-dump SRR3372215 --split-3
    result: SRR3372215_1.fastq and SRR3372215_2.fastq are got;
8. Convert SRR3372215_1.fastq and SRR3372215_2.fastq to FASTA
    command: python SR_fastq2a.py SRR3372215_1.fastq
    python SR_fastq2a.py SRR3372215_2.fastq
    result: SRR3372215_1_out.fasta and SRR3372215_2_out.fasta are got.
9. Concatenate the forward and reverse short reads files;

command: python mergeSR.py SRR3372215_1_out.fasta SRR3372215_1_out.fasta
result: SR.fa is got.

10. Put the SR.fa and LR.fa into Data folder
11. Run LSCplus

command: ./LSCplus
result: corrected_LR.fa and corrected_LR_full.fa were got.

12. Analyze outputs

*: Steps are optional, not necessary
